# Demetallization of *Enterococcus faecalis* biofilm: a preliminary study

**DOI:** 10.1590/1678-7757-2017-0374

**Published:** 2018-01-24

**Authors:** Carlos ESTRELA, Rodrigo COSTA E SILVA, Roberta Cerasi URBAN, Pablo José GONÇALVES, Júlio A. SILVA, Cyntia R.A. ESTRELA, Jesus Djalma PECORA, Ove A. PETERS

**Affiliations:** 1 Universidade Federal de Goiás, Faculdade de Odontologia, Departamento de Ciências Estomatológicas, Goiânia, Goiás, Brasil.; 2 Universidade Federal de Goiás, Instituto de Física, Goiânia, Goiás, Brasil.; 3 Universidade Federal de São Carlos, Instituto de Química, São Carlos, São Paulo, Brasil.; 4 Universidade de São Paulo, Faculdade de Odontologia de Ribeirão Preto, Departamento de Endodontia, Ribeirão Preto, São Paulo, Brasil.; 5 University of the Pacific, Arthur A. Dugoni School of Dentistry, Department of Endodontics, San Francisco, California, USA.

**Keywords:** Biofilms, EDTA, Sodium hypochlorite, Enterococcus faecalis

## Abstract

**Objectives:**

To determine the concentration of calcium, iron, manganese and zinc ions after the application of chelator to *Enterococcus faecalis* biofilms.

**Material and Methods:**

Fifty bovine maxillary central incisors were prepared and inoculated with *E. faecalis* for 60 days. The following were used as irrigation solutions: 17% EDTA (pH 3, 7 and 10), 2.5% sodium hypochlorite (NaOCl) combined with 17% EDTA (pH 3, 7 and 10), distilled water (pH 3, 7 and 10), and 2.5% NaOCl. Each solution was kept in the root canal for five minutes. Fifteen uncontaminated root canals were irrigated with 17% EDTA (pH 3, 7 and 10). Six teeth were used as bacterial control. The number of calcium, iron, manganese and zinc ions was determined using flame atomic absorption spectrometry. Mean ± standard deviation (SD) values were used for descriptive statistics.

**Results:**

Calcium chelation using 17% EDTA at pH 7 was higher than at pH 3 and 10, regardless of whether bacterial biofilm was present. The highest concentration of iron occurred at pH 3 in the presence of bacterial biofilm. The highest concentration of manganese found was 2.5% NaOCl and 17% EDTA at pH 7 in the presence of bacterial biofilm. Zinc levels were not detectable.

**Conclusions:**

The pH of chelating agents affected the removal of calcium, iron, and manganese ions. The concentration of iron ions in root canals with bacterial biofilm was higher after the use of 17% EDTA at pH 3 than after the use of the other solutions at all pH levels.

## INTRODUCTION

The destruction of bacterial biofilm has been a significant topic in endodontic research over the years^13^ ^,^ ^21^ ^,^ ^25^. For this purpose, antibacterial and physicochemical effectiveness of a series of irrigating solutions has been evaluated, including sodium hypochlorite, chlorhexidine, cationic detergent, ethylenediaminetetraacetic acid (EDTA), MTAD, ozonated water, apple vinegar and other solutions^9^ ^-^ ^11^ ^,^ ^15^ ^,^ ^16^ ^,^ ^20^ ^,^ ^26^ ^-^ ^30^ ^,^ ^33^.

Sodium hypochlorite is an irrigating solution largely used because of a combination of several properties: antimicrobial action, tissue dissolution capacity and acceptable biological compatibility at less concentrated solutions^9^ ^-^ ^12^ ^,^ ^15^ ^,^ ^16^ ^,^ ^20^ ^,^ ^29^ ^,^ ^30^. Sodium hypochlorite acts on enzymatic sites essential for bacterial viability, promoting irreversible microbial inactivation due to the action of hydroxyl ions and chloramination. The dissolution of organic tissue occurs during saponification, when sodium hypochlorite degrades fatty acids and lipids, resulting in soap and glycerol^10^.

Root canal preparation produces a smear layer, which is composed of dentin chips, remnants of pulp tissue and odontoblastic processes, microorganisms and chemicals found in irrigating agents^15^ ^,^ ^16^ ^,^ ^26^ ^-^ ^28^ ^,^ ^33^. Ethylenediaminetetraacetic (EDTA) at a neutral pH promotes the chelation of calcium ions in the dentin^15^ ^,^ ^16^ ^,^ ^26^ ^,^ ^27^. This chelating agent is commonly used for smear layer removal, but has a poor antibacterial effect^15^ ^,^ ^16^ ^,^ ^28^. A mixture of a new solution for the removal of the smear layer containing 3% doxycycline hyclate, 4.25% citric acid, and 0.5% Tween 80 (MTAD, Dentsply Sirona, York, PA, USA) has been evaluated as a final rinse of root canal surfaces after preparation. MTAD effectively removed the smear layer when used as a final rinse^28^.

The destruction of bacterial life is dependent on the conditions of their growth and multiplication, among which are physical-chemical factors such as: temperature, pH, osmotic pressure, and oxygen, carbon dioxide and substrate concentrations. The dynamics of endodontic infections suggests the following ecological determinants: oxidation-reduction potential; nutrient availability; and microbial interactions, such as synergism or antagonism^4^ ^-^ ^6^. Bacterial cells require carbon, nitrogen, oxygen, hydrogen, phosphorous, sulphur, iron, sodium, calcium, magnesium and water. Other nutrients are required in extremely low amounts, and are, therefore, called trace nutrients (zinc, copper, manganese, molybdenum and cobalt) that act as enzymatic activators at the level of the cytoplasmic membrane^3^ ^-^ ^6^ ^,^ ^17^ ^,^ ^18^ ^,^ ^23^ ^,^ ^24^ ^,^ ^32^. Bacteria are capable of complex differentiation and behaviors and may easily organize in communities attached to a root canal surface^7^ ^,^ ^8^ ^,^ ^14^ ^,^ ^19^ ^,^ ^22^.

Keogh, et al.^18^ (2017) report a new form of iron-dependent metabolism for *E. faecalis* where, in the absence of heme, respiration components can be used for extracellular electron transfer (EET). Iron augments *E. faecalis* biofilm growth and generates alterations in biofilm matrix, cell spatial distribution, and biofilm matrix properties.

These results suggest that other alternatives to disrupt biofilm, such as the removal of essential chemicals from bacterial biofilm and metabolism, should be investigated. Iron ion is an essential element for bacterial growth and metabolism, and an indispensable cofactor of numerous bacterial biologic processes^4^ ^-^ ^6^ ^,^ ^12^ ^,^ ^24^. Given the importance of iron on bacterial biofilm and metabolism, and the lack of studies about it, this study analyzed a demetallization process by determining the concentration of calcium, iron, manganese and zinc ions after the use of a chelating agent to *Enterococcus faecalis* biofilm samples.

## Material and methods

### Teeth preparation

Seventy-one bovine maxillary central incisors recently extracted and with a closed apical foramen were immersed in 5% sodium hypochlorite (Fitofarma, Lt. 20442, Goiânia, GO, Brazil) for 60 min to remove organic tissues. The teeth were kept in 0.1% thymol solution and under refrigeration. Subsequently, the teeth were retrieved, and the crowns were sectioned at 90 degrees to the long axis of the tooth using Endo-Z burs (Dentsply Maillefer, Ballaigues, Switzerland) in a high-speed handpiece, under continuous water/air spray. The tooth length was standardized to 15 mm (from root apex to coronal border). Canal patency was checked with #20 K-File (Dentsply Maillefer, Ballaigues, Switzerland) introduced into the root canal of each tooth up to the point where it was visualized at the apex. The anatomical diameter of the root canals typically corresponded to a #50 K-file. Root canals were then rinsed with 20 milliliters of deionized water to remove possible dentin chips and then autoclaved for 30 min at 120°C.

### Biofilm formation

*Enterococcus faecalis* (#29212, ATCC Manassas, VI) was inoculated in 7 mL of brain heart infusion (BHI; Difco Laboratories, Detroit, MI, USA) and incubated at 37^o^C for 24 hours. After that, suspensions were prepared on the surface of BHI plates under the same incubation conditions; bacterial cells were resuspended in saline and adjusted to the #1 McFarland turbidity standard (3x10^8^ cells/mL).

Five milliliters of sterilized BHI were mixed with 5 mL of the bacterial inoculum, and the samples were inoculated with *E. faecalis* for 60 days, by using sterilized syringes to fill each root canal. At 72-hour intervals, this process was repeated, always using 24-hour pure cultures prepared and adjusted to the #1 McFarland standard. The teeth were kept under suitable atmospheric conditions and in a humid environment at 37°C. All experimental procedures were carried out under aseptic conditions.

The teeth were randomly allocated to one of four irrigant groups prepared with *E. faecalis* biofilm or one control group with no biofilm, as follows: G1 – 17% EDTA (pH 3, 7 and 10; n=15); G2 – 2.5% sodium hypochlorite and 17% EDTA (pH 3, 7 and 10; n=15); G3 – distilled water (pH 3, 7 and 10; n=15); G4 – 2.5% sodium hypochlorite (pH 11; n=5); and G5 – no biofilm and 17% EDTA (pH 3, 7 and 10; n=15). Six teeth were added and used as controls to test the aseptic control of root canals and bacterial viability during all the experiment. All irrigant solutions were prepared in the research laboratory of the Institute of Chemistry (Federal University of Goiás, Brazil). The 2.5% sodium hypochlorite solution was obtained by dilution of a 12% solution and prepared at pH 11. The deionized water was obtained using the Milli-Q^®^ water purification system (Millipore, Temecula, CA, USA) and also prepared at pH 3, 7 and 10.

### Determination of calcium, iron, manganese, and zinc ion concentrations

The teeth were irrigated with 20 mL of distilled water to remove the excess of the remaining medium, and dried with sterilized absorbent paper points. In the 17% EDTA group, the root canals were completely filled with the chelating agent in pH 3, 7 and 10 using a syringe and a 30-gauge needle (Ultradent Products, South Jordan, UT, USA) shaken for 5 minutes (tube shaker, P56, Araraquara, SP, Brazil). The same irrigation strategy was used for all groups. In the 2.5% sodium hypochlorite and 17% EDTA group, the root canals were first irrigated with 5 mL of 2.5% sodium hypochlorite, dried and then also completely filled with the chelating agents, for 5 minutes. In the 2.5% sodium hypochlorite and deionized water groups, the root canals were irrigated with these solutions, and dried before collecting samples. In order to collect samples from the root canals, the cervical part of the tooth was kept out of another Eppendorf tube, while the root was inside it. Immediately after the application of the test irriganting solutions, all samples were flushed with 7.5 mL deionized water. The total volume collected in the tubes was used to measure chemical element concentrations using atomic absorption spectrophotometry.

The calcium and iron ion concentrations were measured using flame atomic absorption spectrometry (AAnalyst 800 FAAS, Perkin-Elmer Inc., Shelton, CT, USA) and deuterium background correction. A Xenon short-arc lamp, operating at 13 mA, was used as the radiation source. The analyses were performed at 422.7 nm for calcium and 248.3 nm for iron. The acetylene flow rate and the burner height were adjusted to obtain maximum absorbance signal.

Manganese and zinc ion concentrations were measured using graphite furnace atomic absorption spectrometry (GFAAS, AAnalyst 800, Perkin-Elmer INC., Shelton, CT, USA) in a transversely heated graphite tube atomizer and background longitudinal Zeeman effect correction. Hollow cathode lamps of manganese (279.5 nm) and zinc (213.9 nm) were used as radiation sources, both operating at 20 mA. The graphite tube with integrated platforms had a pyrolytic coating. Argon was used as the purge gas. The analyses were performed in triplicate, and spectrometry results were described as mean ± standard deviation (SD) values.

## Results

Table 1 presents the spectrometry data obtained for calcium, iron, manganese and zinc ions released when chelating agents were used. Calcium concentration when EDTA was used at pH 7 was higher than at pH 3 and 10, regardless of presence of bacterial biofilm. The highest concentration of iron ions was at pH 3 in the presence of bacterial biofilm (isolated or after the use of NaOCl). The highest concentration of manganese ions was found for NaOCl and EDTA at pH 7 in the presence of bacterial biofilm. On the other hand, zinc ions were not detected because their concentration was lower than the detection limits of the method used. The substances without chelating characteristics (NaOCl and deionized water) did not show any ionic release.

**Table 1:** Mean±standard deviation (SD) values of concentration of chemical elements detected after the use of chelating agents at different pH levels on *E. faecalis* biofilm

*E. faecalis* biofilm	Irrigating solutions	pH	Calcium (mg/L)	Iron (mg/L)	Manganese (mg/L)	Zinc (mg/L)
Yes	17% EDTA (n=15)	3	929.0±52.3	18.40±0.45	96.6±4.7	<LOD
		7	1968.3±141.4	8.01±0.86	117.9±4.2	<LOD
		10	921.9±56.6	2.27±0.10	70.4±3.1	<LOD
Yes	2.5% NaOCl + 17% EDTA (n=15)	3	796.1±87.7	18.45±0.86	101.4±4.2	<LOD
		7	1124.2±93.3	<LOQ	213.5±4.7	<LOD
		10	725.4±38.2	16.56±0.86	93.9±7.4	<LOD
Yes	Deionized water (n=15)	3	<LOD	<LOD	0.8±0.2	<LOD
		7	<LOD	<LOD	<LOQ	<LOD
		10	<LOD	<LOD	<LOQ	<LOD
Yes	2.5% NaOCl (n=5)	11	2.5±0.2	<LOQ	<LOQ	<LOD
No	17% EDTA (n=15)	3	673.1±56.6	2.00±0.11	208.7±3.1	<LOD
		7	2036.2±101.8	0.40±0.03	72.5±2.6	<LOD
		10	790.4±60.8	1.22±0.07	70.9±3.6	<LOD

(LOQ – limit of quantification; LOD – limit of detection; Calcium – LOQ = 1.0, LOD = 0.3, SD = 0.00017;

Iron – LOQ – 0.3, LOD 0.1, SD = 0.00044; Manganese – LOQ = 0.7, LOD = 0.2, SD = 0.0005)

(Six teeth were used as microbial control, 3 as positive controls and 3 as negative controls)

## Discussion

Intraradicular microorganisms are considered the main cause of persistent periapical periodontitis. Various strategies to disrupt the intracanal biofilm have been evaluated^11^ ^,^ ^13^ ^,^ ^21^ ^,^ ^25^. Planktonic microorganisms are susceptible to appropriate clinical protocols^11^ ^,^ ^21^ ^,^ ^25^. However, the presence of biofilm remains an obstacle to the success of root canal treatments^2^ ^,^ ^13^ ^,^ ^21^ ^,^ ^25^.

Currently, no strategy has proved to be effective in eradicating bacterial biofilms. An alternative may be to use chelating agents to remove chemical elements, essential for bacterial metabolism and, hence, disrupt biofilms. In this preliminary study, EDTA in acid, neutral and basic solutions (pH 3, 7 and 10) was used to determine some important cationic ions release from *E. faecalis* biofilm. The results showed that pH changed ion concentrations in biofilm. The concentration of iron ions in the root canals was higher in the groups with 17% EDTA at pH 3 than in the groups with other solutions and different pH levels in the presence of biofilm. The higher calcium concentration was found in the 17% EDTA group at pH 7, regardless of biofilm presence.

In the group of 2.5% sodium hypochlorite associated with 17% EDTA (in pH 7), a lower concentration of calcium ions was observed compared to an isolated solution of 17% EDTA with or without bacterial biofilm. One possible explanation may be an interaction between these substances. Zehnder, et al.^32^ (2005) analyzed interactions of EDTA and citric acid with sodium hypochlorite. The chelation reaction during root canal irrigation was not necessarily an equilibrium reaction determined by a standard stability constant, because rate effects and ligand exchange reactions might considerably affect complex formation.

The action of chelating agents on the bacterial biofilm implies in the same ionic complexation of metals present in dentin, and especially with the calcium ions compared with iron ions. It was not possible to distinguish the calcium ions between the groups with and without bacterial biofilm. A different event was identified with the iron ions, in which a higher presence of this ion was identified in the groups with biofilm compared to the absence of bacterial biofilm.

The importance of iron on bacterial biological processes has been demonstrated in previous studies^3^ ^-^ ^6^ ^,^ ^17^ ^,^ ^18^ ^,^ ^23^ ^,^ ^24^ ^,^ ^33^. The acquisition of metal ions by all microorganisms is indispensable for survival in the environment or in their infected host. Iron is an essential nutrient for bacterial growth and for various metabolic and enzymatic processes because of its role as metalloprotein components, cofactors, facilitator of enzymatic catalysis and element that maintains chemical gradients across cell membranes^4^ ^-^ ^6^ ^,^ ^18^ ^,^ ^23^ ^,^ ^24^.

Iron is essential for bacterial cell metabolism. Strict aerobe and facultative bacteria under aerobic conditions excrete small quantities of chelated complexes with iron (siderophores, iron carriers); this compound is taken up by specific receptors on the cell surface, and the essential nutritive molecule is then released inside the bacteria. In anaerobiotic environments, iron is highly soluble and is used as other metal ions. Another probable iron acquisition strategy of bacteria is the production of hemolysins, which lyse erythrocytes and subsequently leads to the release of hemoglobin, a potential source of iron for bacterial metabolism. Nutritive variables depend on the origin of a specific substance, its chemical composition and the amount required by a specific microorganism^4^. Porcheron, et al.^24^ (2013) reported that iron is the most abundant transition metal in hosts, but free ferrous iron (Fe2+) is extremely rare. A strategy that has been called nutritional immunity may reduce the risk of infection by preventing pathogens from acquiring iron. As these metals are essential cofactors for bacterial physiology and growth, it is not surprising that metal transporters are associated in the virulence of pathogenic enterobacteria. Other trace minerals (zinc and manganese) may also be sequestered to protect against invading pathogens^17^; however, as for Zn2+, the mechanisms of Mn2+ transport across the outer membrane have not yet been defined for enterobacteria^23^. Wakeman and Skaar^31^ (2012) reported that metal ion fluctuations are used as a tool to kill invading pathogens. Metal ion homeostasis in Gram-positive pathogens determines metal regulated virulence factors and metabolic processes that are critical for the survival of invading microorganisms and may ultimately yield novel drug targets.

In this study, calcium ions concentrations were higher in neutral EDTA solutions (pH 7) than in acid or alkaline EDTA solutions (pH 3 or 10), regardless of the presence of bacterial biofilm (Table 1). Serper and Çalt^26^ (2002) showed that EDTA effectively demineralizes dentin depending on the concentration and pH of EDTA, which was more effective on dentin demineralization at a neutral pH (7.5) than when applied at pH 9.0. Spanó, et al.^27^ (2009) determined the concentration of calcium ions and smear layer removal by using root canal chelators and found that 15% EDTA solutions removed the highest concentration of calcium ions, followed by 10% citric acid, when compared with 10% sodium citrate, apple vinegar, 5% acetic acid, and 5% malic acid. Smear layer removal was the most efficient when 15% EDTA and 10% citric acid were used.

The increase of calcium ion concentrations may also play an important role in bacterial biofilm formation^14^ ^,^ ^19^. George and Kishen^14^ (2005) studied the ability of *E. faecalis* to develop biofilm under aerobic, anaerobic, nutrient-rich and nutrient-deprived conditions. *E. faecalis* grown in an aerobic nutrient-rich environment produced irregularly shaped amorphous biofilm macro structures measuring 500 to 1000 μm. These structures were found to be bacterial cell aggregates. Under nutrient-rich conditions, an increased concentration of calcium (Ca) and phosphorus (P) was observed, but the Ca/P ratio was similar to that of dentine. Kishen, et al.^19^ (2006) found a different sequence for the interaction of *E. faecalis* with dentin: 1 – *E. faecalis* formed biofilm on the root canal dentin; 2 – bacteria induced dissolution of the mineral fraction in the dentin substrate; 3 – a reprecipitated apatite layer was formed in the biofilm. The authors mentioned that this ability of *E. faecalis* to form such calcified biofilm on root canal dentin might contribute to their persistence after endodontic treatment. Other authors^20^ found that the presence of a smear layer reduced that antimicrobial activity of 2.5% sodium hypochlorite.

Another recent alternative to destroy *E. faecalis* biofilms, suggested by Almeida, et al.^1^ (2016) was the use of 17% EDTA and a modified salt solution (MSS). The MSS was prepared by dissolution of sodium chloride and potassium sorbate in demineralized water. EDTA detached most cells from biofilms, but had a minor antimicrobial effect. In addition to a great antimicrobial effect, MSS also detached 94% of biofilm cells.

The methodology used in this study (atomic absorption spectrophotometry) to measure chemical element concentrations of the test solutions was analyzed as described previously^27^. The bacteria selected play an important role in root canal infections^1^ ^,^ ^8^ ^,^ ^9^ ^,^ ^14^ ^,^ ^18^ ^,^ ^30^. The period of 60 days to root canal contamination is sufficient for the bacteria to infect and to form biofilm on the root canal surface^3^ ^,^ ^11^.

The traces of iron ions detected in the samples with biofilm suggest that EDTA at pH 3 may be an important complexation agent to remove iron. The complexation of iron ions using chelating agents in bacterial biofilm may determine new directions to an old problem, root canal infection. The removal of calcium and iron from bacterial biofilms may result in new approaches to antibiofilm strategies based on demetallization. Biofilm models (abiotic, biotic; monospecies, multispecies; young, mature), biological indicators, and types, concentrations, time of action and pH of the irriganting solutions and chelating agents may determine distinct antibacterial potentials. Thus, further studies should be taken to confirm the hypothesis of the effect of ionic chelation on the destruction of bacterial biofilm.

## Conclusions

The pH of chelating agents affected the removal of calcium, iron, and manganese ions. The concentration of iron ions in the biofilm in root canals was higher in the groups with 17% EDTA solution at pH 3 than in the groups with other solutions at other pH.
